# Airway clearance techniques for adults receiving extracorporeal membrane oxygenation for severe acute respiratory failure: a scoping review protocol

**DOI:** 10.1136/bmjopen-2025-111032

**Published:** 2025-11-19

**Authors:** Tom Lunn, Ema Swingwood, Hannah Cochrane, Steven Walker, Fiona Cramp

**Affiliations:** 1 Diagnostics and Therapies, University Hospitals Bristol and Weston NHS Foundation Trust, Bristol, UK; 2 Bristol Biomedical Research Centre, University of Bristol, Bristol, UK; 3 Centre for Health and Clinical Research, University of the West of England, Bristol, UK; 4 Library and Information Services, University Hospitals Bristol and Weston NHS Foundation Trust, Bristol, UK; 5 North Bristol NHS Trust, Westbury on Trym, UK

**Keywords:** Adult intensive & critical care, Pulmonary Disease, Physical Therapy Modalities, Respiratory Therapy

## Abstract

**Abstract:**

**Introduction:**

Patients receiving extracorporeal membrane oxygenation (ECMO) for severe acute respiratory failure (SARF) often experience significant challenges with airway clearance due to disease severity, ultra-protective ventilation strategies and suppression of mucociliary and cough mechanisms. Extremely low tidal volumes and minimal flow rates further hinder secretion mobilisation. Despite increasing global use of ECMO, there is currently no synthesis of evidence describing airway clearance practices, their physiological rationale or outcomes in this population.

This scoping review aims to explore the extent and nature of evidence on airway clearance interventions in invasively ventilated adult patients receiving ECMO for SARF. Specific objectives include mapping the types of interventions described and specific parameters, their intended physiological effects, reported outcomes and safety considerations.

**Methods and analysis:**

This scoping review will be conducted in accordance with the Joanna Briggs Institute methodology and reported using Preferred Reporting Items for Systematic Reviews and Meta-Analyses extension for Scoping Reviews guidelines. A comprehensive search will be undertaken across Embase, Ovid Emcare, Ovid Medline, CINAHL and grey literature sources including TRIP and Google Scholar. Eligible studies will include original data on airway clearance interventions in adult patients (≥16 years) receiving ECMO for SARF. Studies focused on paediatric populations, extracorporeal carbon dioxide removal, or solely cardiac ECMO will be excluded.

Two independent reviewers will screen titles, abstracts and full texts, extract data using a piloted tool and summarise findings using descriptive statistics and narrative synthesis. Basic qualitative content analysis will support mapping of interventions including parameters, their physiological and clinical rationale, and reported outcomes including adverse effects.

**Ethics and dissemination:**

As this study involves a review of existing literature and does not include collection of primary data, ethical approval is not required. Findings will be disseminated through publication in a peer-reviewed journal and presentation at relevant academic and professional meetings. The results are expected to highlight variations in practice and provide a foundation for future research aimed at optimising respiratory care and improving outcomes for patients receiving ECMO for SARF.

**Registration:**

osf.io/ptfr7.

**STRENGTHS AND LIMITATIONS OF THIS STUDY**

- This scoping review will be conducted in accordance with the Joanna Briggs Institute methodology, ensuring methodological rigour and transparency.
- The study explores an underexamined aspect of critical care physiotherapy: secretion clearance strategies in patients receiving extracorporeal membrane oxygenation for acute respiratory failure, where severely reduced tidal volumes and low expiratory airflow present a unique clinical challenge.
- A comprehensive and systematic search strategy, developed in consultation with an information specialist, will be used across multiple databases to maximise the retrieval of relevant literature.
- The review will include a broad range of study designs and grey literature to capture the diversity of clinical practice and emerging techniques.
- This study will not include formal quality appraisal of included evidence, nor assess the effectiveness or safety of interventions.

## Background

 Secretion clearance is a fundamental component of respiratory care in critically ill patients, and it presents distinct challenges in those receiving extracorporeal membrane oxygenation (ECMO) for severe acute respiratory failure (SARF). ECMO, in this setting, serves as an advanced life support modality that provides gas exchange for patients with SARF who are unresponsive to conventional mechanical ventilation.

In patients receiving ECMO, the combination of profound lung pathology, ultra-lung protective ventilation strategies, deep sedation and neuromuscular blockade significantly impairs normal airway clearance mechanisms, including mucociliary transport and the cough reflex.^1^ Impaired clearance can lead to lung volume loss, worsening ventilation-perfusion mismatch, atelectasis and increased susceptibility to ventilator-associated pneumonia (VAP), with secretion retention recognised as a key contributing factor to infection development.^2^ VAP is associated with increased mortality, prolonged mechanical ventilation and extended intensive care unit stays, making the prevention and management of secretion accumulation a priority in critical care.

Secretion clearance refers to the physiological and therapeutic processes aimed at mobilising and removing mucus and other airway secretions to maintain airway patency, optimise ventilation-perfusion matching and reduce the risk of infection. Under normal conditions, this process relies on an intact mucociliary escalator and effective cough reflex to transport secretions from the distal airways to the oropharynx for expectoration or swallowing. In mechanically ventilated patients, these mechanisms are frequently compromised, necessitating additional interventions. These may include lung hyperinflation, suctioning, patient positioning, manual techniques such as percussion and vibration, and the use of mucolytic agents.^3^ Each of these strategies aims to mobilise secretions, enhance the efficacy of airway suctioning and minimise associated complications.

While secretion management strategies are well established in mechanically ventilated patients with normal tidal volumes,^3^ these approaches have not been specifically evaluated in people receiving ECMO, where tidal volumes are frequently minimal, and respiratory mechanics are profoundly altered. ECMO use presents unique challenges, including the concept of the ‘baby lung’ (low functional lung capacity),^4^ heterogeneous time constants and the requirement to balance minimal ventilator settings with effective secretion clearance strategies. Traditional techniques for secretion mobilisation may not directly translate to this context, and interventions must be carefully adapted to avoid compromising lung protection or ECMO circuit dynamics.

To date, there is an absence of a comprehensive synthesis of the evidence regarding secretion clearance in people receiving invasive mechanical ventilation (IMV) and ECMO. Furthermore, there are no evidence-based national guidelines in the UK.

### Objective

The primary objective of this review is to identify which airway clearance interventions are currently being used in patients receiving IMV and ECMO for SARF, how they are being applied, and the physiological and clinical rationales underpinning their use.

The scoping review will also aim to address the following secondary objectives:

- What are the intervention parameters (eg, timing, dosage), and how do these align with different stages of the ECMO patient pathway?
- What outcomes are used to evaluate the effects of airway clearance interventions (eg, secretion volume, oxygenation indices, lung compliance, ventilation–perfusion matching, physiological response)?
- How are safety and adverse effects monitored and managed during airway clearance interventions?
- Which health professionals deliver the interventions, and what specific training or competencies are required (eg, physiotherapists, nurses, respiratory therapists, medical staff)?

## Method

### Study design

A scoping review was selected to address the study questions and is considered appropriate due to the complex evidence base including the potential heterogeneity of primary research data and need to map the extent, range and nature of available research rather than to answer a narrowly defined question.^5^ This methodology enables a comprehensive overview of existing research, highlights uncertainty and identifies priorities for future inquiry.

This scoping review will be conducted in accordance with the Joanna Briggs Institute (JBI) methodology for scoping reviews^5^ and reported in line with the Preferred Reporting Items for Systematic Reviews and Meta-Analyses extension for Scoping Reviews (PRISMA-ScR).^6^ Following this approach will help to ensure a rigorous, transparent and reproducible process for study selection, data extraction and synthesis.

### Protocol and registration

This scoping review was registered on the Open Science Framework on the 1 September 2025 Unique identification number: osf.io/ptfr7

### Patient and public involvement

Individuals with lived experience of intensive care, including those who had undergone ECMO, contributed to this review through a group patient and public involvement and engagement (PPIE) meeting. During the session, they endorsed the importance of the topic and helped refine the review focus. They will also be invited to provide feedback on the preliminary findings to support interpretation and dissemination.

### Eligibility criteria

Inclusion criteria for the review were identified using the Population; Concept; Context framework for scoping reviews in accordance with the JBI methodology framework.^5^

### Population

This scoping review will focus exclusively on the adult population receiving IMV and ECMO. Therefore, studies will be included only if the patient population is aged ≥16 years or where results are reported separately for adults.

Studies will be included if they focus on patients receiving ECMO for respiratory failure. This may encompass those initially managed with ultra-lung protective ventilation using very low tidal volumes, as well as patients in recovery phases marked by improving pulmonary compliance and the return of spontaneous ventilation. Studies exclusively involving patients receiving extracorporeal support for primary cardiac failure will be excluded, as they do not reflect the target population with primary respiratory pathology.

Paediatric studies will be excluded due to fundamental anatomical and physiological lung differences. These include smaller and more compliant airways, increased chest wall compliance, ongoing alveolar development, immature respiratory musculature and differing disease aetiology. These factors significantly alter respiratory pathophysiology and response to interventions, rendering findings in paediatric populations non-transferable to adults.

Studies involving patients supported solely by extracorporeal carbon dioxide removal (ECCO₂R) will also be excluded, as ECCO₂R is typically used in the context of isolated hypercapnic respiratory failure and does not reflect the population of interest.

### Concept

This review will include studies that evaluate interventions aimed at airway clearance techniques in people receiving ECMO, delivered by clinicians from any professional discipline (eg, Physiotherapist, Nurse or Doctor).

Airway clearance refers to the mobilisation and removal of pulmonary secretions with the intent to improve respiratory mechanics, restore or maintain lung volumes and optimise ventilation-perfusion (V/Q) matching.

Interventions may include, but are not limited to, manual or mechanical physiotherapy techniques, suctioning methods, ventilator strategies, pharmacological adjuncts (eg, mucolytics), positioning approaches and bronchoscopy.

### Context

This review will be situated within the context of severe respiratory failure managed in the critical care environment. Specifically, it will focus on patients receiving ECMO for life-threatening hypoxaemia. These clinical scenarios are frequently complicated by sepsis, cardiovascular instability and other factors that challenge the safe and effective delivery of routine respiratory care interventions.

### Identifying relevant studies

The search strategy will aim to locate both published and unpublished studies via a three-step search strategy. First, an initial limited search of Embase (Ovid) and Google Scholar was undertaken to identify articles on the topic. The second component will be a comprehensive search strategy based on the text words contained in the titles and abstracts of relevant articles, and the index terms used to describe the articles. The draft search strategy for Embase (Ovid) was submitted for peer review by a second librarian in accordance with the Peer Review of Electronic Search Strategies guidelines.^7^ The peer-reviewed search strategy is provided as online supplemental additional file 1. This search strategy will be customised for each database, including MEDLINE (Ovid), Emcare (Ovid) and CINAHL (EBSCOhost). Step three will ensure searching of grey literature using a modified search strategy on TRIP and Google Scholar. We will consider search results from the first 10 pages of TRIP and Google Scholar for inclusion in the review. Inclusion and exclusion criteria are shown in table 1. Non-English language publications will be excluded due to resource limitations. Studies published in English from 2000 onwards will be included. A 25-year review period has been selected because the clinical use of ECMO for respiratory failure expanded substantially following the 2009 H1N1 influenza pandemic, which underscored ECMO’s efficacy in managing severe acute respiratory distress syndrome.

**Table 1:** Inclusion and exclusion criteria for studies

Inclusion	Exclusion
Patients receiving airway clearance techniques	Children (<16 years old)
Adults (≥16 years old)	Patients supported solely by extracorporeal carbon dioxide removal
Patients receiving ECMO for severe acute respiratory failure	Patients exclusively receiving extracorporeal support for primary cardiac failure
Patients receiving invasive mechanical ventilation	
Any study design, including but not limited to: Controlled trials Before-and-after studies Interrupted time-series studies	
Additional sources of information: Expert opinion pieces Policies and guidelines Healthcare standards Technical reports Conference proceedings Published abstracts	
Published from 2000 onwards	

ECMO, extracorporeal membrane oxygenation.

### Selection of studies

Following the search, all identified citations will be collated and uploaded into Rayyan (a web-based review management platform) and duplicates removed. Titles and abstracts will be screened by two or more independent reviewers for assessment against the inclusion criteria for the review. Where one or more reviewers identify a citation as potentially relevant, sources will be retrieved and imported into Rayyan. The full text of selected citations will be assessed in detail against the inclusion criteria by two independent reviewers. Reasons for exclusion of sources of evidence at full text, that do not meet the inclusion criteria, will be recorded and reported in the scoping review. Any disagreements that arise between the reviewers at full text stage will be resolved through discussion, or with an additional reviewer.

### Data charting process

Data will be extracted from sources included in the scoping review by two or more independent reviewers using a data extraction tool developed by the reviewers. The data extracted will include specific details about the participants, concept, context, study methods and key findings relevant to the review questions as per the JBI Manual of Evidence Synthesis V.10.2.7.^5^

The draft data extraction tool will be modified and revised during the process of extracting data from each included evidence source (see table 2). Modifications will be detailed in the scoping review. Any disagreements that arise between the reviewers will be resolved through discussion, or with an additional reviewer/s. If necessary, authors will be contacted to request missing or additional data.

**Table 2:** Data extraction table

Variable	Description of what will be documented
Authors	Full names of the authors of the included studies
Title	Full title of each included publication
Publication year	Year in which the study was published or made publicly available
Objective(s)	A description of the primary aim(s) or objective(s)
Study design (where relevant)	Methodological approach used (eg, RCT, observational study, case study)
Country	Country where the study was conducted/experts were based
Population characteristics	Description of the population, including demographics (eg, age, sex/gender), underlying pathology (eg, ARDS, asthma), type of ECMO used (eg, VV-ECMO, VA-ECMO) and type of invasive mechanical ventilation (if provided)
Intervention(s) and prescription details	Description of the intervention(s), including type, timing, frequency and duration
Rationale	Stated physiological rationale for use of intervention(s)
Clinician profession	Professional role of the individual(s) delivering the intervention (eg, physiotherapist, nurse, doctor). Staffing requirement for the delivered intervention (if provided)
Outcome evaluated	Outcome measures used to assess intervention effectiveness (eg, secretion volume, oxygenation indices, lung compliance)
Risks and safety considerations	Any risks, adverse effects or safety considerations reported in relation to the intervention

ARDS, acute respiratory distress syndrome; ECMO, extracorporeal membrane oxygenation; RCT, randomised controlled trial; VA, veno-arterial ; VV, veno-venous.

### Analysis and presentation of findings

The results of the search and the study inclusion process will be reported in full in the final scoping review and presented in a PRISMA flow diagram.^8^ Findings will be mapped by descriptive statistics (frequency counts) and presented using visualisation techniques (eg, tables, charts and/or figures) as appropriate. A narrative summary will accompany quantitative results. Basic qualitative content analysis will be conducted to identify key characteristics of the interventions used, plus the rationale, frequency, safety assessment and outcomes recorded. Reviewers will take an inductive approach to content analysis to allow better representation of these primary and secondary objectives of the scoping review.

### Amendments

The study will adhere to the protocol as planned, with regular team-wide progress reviews. Any deviations or amendments made during the review will be documented and clearly reported in the final publication.

## Ethics and dissemination

Ethical approval was not required as this study represents a protocol for a scoping review and does not involve human participants. Local research governance oversight and support were provided by the Research and Development Department, University Hospitals Bristol and Weston NHS Foundation Trust (research@uhbw.nhs.uk). Findings will be disseminated through publication in a peer-reviewed journal and presentation at relevant academic and professional meetings. The results are expected to highlight variations in practice and provide a foundation for future research aimed at optimising respiratory care and improving outcomes for patients receiving ECMO for SARF.

## Supplementary material

10.1136/bmjopen-2025-111032 online supplemental file 1
