# Supplementary material for: Airway clearance techniques for adults receiving extracorporeal membrane oxygenation for severe acute respiratory failure: a scoping review protocol
Source: BMJ Open. 2025 Nov 19;15(11):e111032. doi: 10.1136/bmjopen-2025-111032 (PMC12636914; doi:10.1136/bmjopen-2025-111032)
Supplement: online supplemental file 1 [file bmjopen-15-11-s001.docx]

**Additional File No. 1: Search Strategy**

**Airway clearance techniques for adults receiving extracorporeal membrane oxygenation for severe acute respiratory failure: A scoping review protocol**

**Medline**

| 1 | Extracorporeal Membrane Oxygenation/ |
| --- | --- |
| 2 | ("extra?corporeal oygenation*" or "Extra?corporeal Membrane Oxygenat*" or "?ECMO" or “VV?ECMO” or “ECLS”).ti,ab,kw. |
| 3 | or/1-2 |
| 4 | Respiratory Therapy/ |
| 5 | Breathing Exercises/ |
| 6 | Bronchoscopy/ |
| 7 | Bronchoscop*.ti,ab,kw. |
| 8 | ((respirat* or breathing or chest or pulmonary) adj3 (therap* or physiotherap* or exercise* or control)).ti,ab,kw. |
| 9 | ((secretion* or mucus or sputum or airway* or chest or lung) adj2 clear*).ti,ab,kw. |
| 10 | (cough* adj3 (control* or technique* or manoeuvr* or maneuver* or assist*)).ti,ab,kw. |
| 11 | mucociliary clearance/ |
| 12 | (mucociliary adj (transport or clear* or escalat*)).ti,ab. |
| 13 | or/4-12 |
| 14 | 3 and 13 |
| 15 | Infant, Newborn/ |
| 16 | (neonate* or NICU or newborn or baby or babies or ((premature or preterm) adj infant*)).ti,ab. |
| 17 | 15 or 16 |
| 18 | 14 not 17 |
| 19 | limit 18 to yr="2000 -Current" |

**EMCARE**

| 1 | extracorporeal oxygenation/ |
| --- | --- |
| 2 | ("extra?corporeal oygenation*" or "Extra?corporeal Membrane Oxygenat*" or "?ECMO" or “VV?ECMO” or “ECLS”).ti,ab,kw. |
| 3 | or/1-2 |
| 4 | Respiratory Therapy/ |
| 5 | breathing exercise/ |
| 6 | Bronchoscopy/ |
| 7 | Bronchoscop*.ti,ab,kw. |
| 8 | ((respirat* or breathing or chest or pulmonary) adj3 (therap* or physiotherap* or exercise* or control)).ti,ab,kw. |
| 9 | ((secretion* or mucus or sputum or airway* or chest or lung) adj2 clear*).ti,ab,kw. |
| 10 | (cough* adj3 (control* or technique* or manoeuvr* or maneuver* or assist*)).ti,ab,kw. |
| 11 | mucociliary clearance/ |
| 12 | (mucociliary adj (transport or clear* or escalat*)).ti,ab. |
| 13 | or/4-12 |
| 14 | 3 and 13 |
| 15 | Infant, Newborn/ |
| 16 | (neonate* or NICU or newborn or baby or babies or ((premature or preterm) adj infant*)).ti,ab. |
| 17 | 15 or 16 |
| 18 | 14 not 17 |
| 19 | limit 18 to yr="2000 -Current" |

**EMBASE**

| 1 | extracorporeal oxygenation/ |
| --- | --- |
| 2 | ("extra?corporeal oygenation*" or "Extra?corporeal Membrane Oxygenat*" or "?ECMO" or “VV?ECMO” or “ECLS”).ti,ab,kw. |
| 3 | or/1-2 |
| 4 | Respiratory Therapy/ |
| 5 | breathing exercise/ |
| 6 | Bronchoscopy/ |
| 7 | Bronchoscop*.ti,ab,kw. |
| 8 | ((respirat* or breathing or chest or pulmonary) adj3 (therap* or physiotherap* or exercise* or control)).ti,ab,kw. |
| 9 | ((secretion* or mucus or sputum or airway* or chest or lung) adj2 clear*).ti,ab,kw. |
| 10 | (cough* adj3 (control* or technique* or manoeuvr* or maneuver* or assist*)).ti,ab,kw. |
| 11 | mucociliary clearance/ |
| 12 | (mucociliary adj (transport or clear* or escalat*)).ti,ab. |
| 13 | or/4-12 |
| 14 | 3 and 13 |
| 15 | Infant, Newborn/ |
| 16 | (neonate* or NICU or newborn or baby or babies or ((premature or preterm) adj infant*)).ti,ab. |
| 17 | 15 or 16 |
| 18 | 14 not 17 |
| 19 | limit 18 to yr="2000 -Current" |

**CINAHL**

| #  S1 | Query  (MH "Extracorporeal Membrane Oxygenation") | | | | Limiters/Expanders  Expanders - Apply equivalent subjects Search modes - Proximity |
| --- | --- | --- | --- | --- | --- |
| S2 | XB (("extra?corporeal oygenation*" or "Extra? corporeal Membrane Oxygenat*" or "?ECMO" or "?ECMO" or “VV?ECMO” or “ECLS”)) | | | | Expanders - Apply equivalent subjects Search modes - Proximity |
| S3 | S1 OR S2 | | | | Expanders - Apply equivalent subjects Search modes - Proximity |
| S4 | (MH "Respiratory Therapy") | | | | Expanders - Apply equivalent subjects Search modes - Proximity |
| S5 | (MH "Breathing Exercises") | | | | Expanders - Apply equivalent subjects Search modes - Proximity |
| S6 | (MH "Bronchoscopy") | | | | Expanders - Apply equivalent subjects Search modes - Proximity |
| S7 | XB bronchoscop* | | | | Expanders - Apply equivalent subjects Search modes - Proximity |
| S8 | | XB (((respirat* or breathing or chest or pulmonary or lung) N3 (therap* or physiotherap* or exercise* or control))) | | Expanders - Apply equivalent subjects Search modes - Proximity | |
| S9 | | XB (((secretion* or mucus or sputum or airway* or chest) N2 clear*)) | | Expanders - Apply equivalent subjects Search modes - Proximity | |
| S10 | | XB ((cough* N2 (control* or technique* or manoeuvr* or maneuver* or assist*))) | | Expanders - Apply equivalent subjects Search modes - Proximity | |
| S11 | | (MH "Mucociliary Clearance") | | Expanders - Apply equivalent subjects Search modes - Proximity | |
| S12 | | XB ((mucociliary N1 (transport or clear* or escalat*))) | | Expanders - Apply equivalent subjects Search modes - Proximity | |
| S13 | | S4 OR S5 OR S6 OR S7 OR S8 OR S9 OR S10 OR S11 OR S12 | | Expanders - Apply equivalent subjects Search modes - Proximity | |
| S14 | | S3 AND S13 | | Expanders - Apply equivalent subjects Search modes - Proximity | |
| S15 | | (MH "Infant, Newborn") | | Expanders - Apply equivalent subjects Search modes - Proximity | |
| S16 | | XB ((neonate* or NICU or newborn or baby or | | Expanders - Apply equivalent subjects | |
|  | | babies or ((prematur preterm) N1 infant*)) | e or Search modes - Proximity  ) | | |
| S17 | | S15 OR S16 | Expanders - Apply equivalent subjects Search modes - Proximity | | |
| S18 | | s14 not s17 | Expanders - Apply equivalent subjects Search modes - Proximity | | |
| S19 | | s14 not s17 | Limiters - Publication Date: 20000101-  20251231  Expanders - Apply equivalent subjects Search modes - Proximity | | |

**Grey Literature Search:**

**TRIP -** [Trip Medical Database](https://www.tripdatabase.com/Home)

| 1 | (title:"extracorporeal membrane oxygenation") |
| --- | --- |
| 2 | (title:"ecmo") |
| 3 | ((title:"ecmo") OR (title:"extracorporeal membrane oxygenation")) |
| 4 | (title:"Breathing Exercise") |
| 5 | (title:"Bronchoscopy") |
| 6 | (title:"cough") |
| 7 | (title:"secretion") |
| 8 | (title:"sputum") |
| 9 | (title:"mucus") |
| 10 | (title:"airway") |
| 11 | (title:"chest") |
| 12 | (title:"physiotherapy") |
| 13 | (title:"respiratory therapy") |
| 14 | ((title:"Breathing Exercise") OR (title:"respiratory therapy") OR (title:"physiotherapy") OR (title:"chest") OR (title:"airway") OR (title:"mucus") OR (title:"sputum") OR (title:"secretion") OR (title:"cough") OR (title:"Bronchoscopy")) |
| 15 | (((title:"Breathing Exercise") OR (title:"respiratory therapy") OR (title:"physiotherapy") OR (title:"chest") OR (title:"airway") OR (title:"mucus") OR (title:"sputum") OR (title:"secretion") OR (title:"cough") OR (title:"Bronchoscopy")) AND ((title:"ecmo") OR (title:"extracorporeal membrane oxygenation"))) |
|  | (title:"neonate") |
|  | ((((title:"Breathing Exercise") OR (title:"respiratory therapy") OR (title:"physiotherapy") OR (title:"chest") OR (title:"airway") OR (title:"mucus") OR (title:"sputum") OR (title:"secretion") OR (title:"cough") OR (title:"Bronchoscopy")) AND ((title:"ecmo") OR (title:"extracorporeal membrane oxygenation"))) NOT (title:"neonate")) |
| 16 | Date Range 2000-2025 |

Google Scholar - [Google Scholar](https://scholar.google.co.uk/?inst=8205494449466756325)

| 1 | ("Extracorporeal Membrane Oxygenation" OR ECMO) AND  ("airway clearance" OR "chest physiotherapy" OR "respiratory therapy" OR "bronchoscopy" OR "secretion clearance" OR "breathing exercises" OR "mucociliary clearance" OR "cough assist") |
| --- | --- |
| 2 | Date Range 2000-2025 |
