# Supplementary material for: Airway clearance techniques for adults receiving extracorporeal membrane oxygenation for severe acute respiratory failure: a scoping review protocol
Source: BMJ Open. 2025 Nov 19;15(11):e111032. doi: 10.1136/bmjopen-2025-111032 (PMC12636914; doi:10.1136/bmjopen-2025-111032)
Supplement: Supplementary file 2 [file bmjopen-2025-111032.reviewer_comments.pdf]

PEER REVIEW HISTORY

BMJ Open publishes all reviews undertaken for accepted manuscripts. Reviewers are asked to complete a checklist review form and are provided with free text boxes to elaborate on their assessment. These free text comments are reproduced below.

ARTICLE DETAILS

Title (Provisional)

Airway clearance techniques for adults receiving extracorporeal membrane oxygenation for severe acute respiratory failure: A scoping review protocol

Authors

Lunn, Tom; Swingwood, Ema; Cochrane, Hannah; Walker, Steven; Cramp, Fiona

---

VERSION 1 - REVIEW

Reviewer 1

Name Ogura, Takayuki

Affiliation Imperial Foundation SAISEIKAI, Utsunomiya Hospital, Tochigi, JAPAN, Emergency Medicine & Critical Care Medicine

Date 23-Sep-2025

COI None

Thank you very much for giving me this opportunity to review this protocol paper about airway clearance interventions in ECMO patients. This study objective is unique and no one has a clear answer for your clinical question.

However, before the study intiation, you have to re-consider about followings.

1. Please clarify whether the person intervening is a doctor or another medical professional.

2. Please consider interventions that can be performed by doctors (For example, bronchoscopy-assisted suction) and those that can be performed by non-doctors (For example, squeezing by a physical therapist).

3. Please distinguish whether the procedure was performed at an ECMO center that is thought to have an abundance of man-power or not (There is data that shows that ECMO treatment at ECMO centers improves patient outcomes, but this is said to be due to sufficient manpower allocation and the accumulation of sufficient case experience.).

I am really looking forward to reviewing your revised protocol paper.

Reviewer 2

Name Belletti, Alessandro

Affiliation IRCCS San Raffaele Scientific Institute, Department of Anaesthesia and Intensive Care

Date 09-Oct-2025

COI None

The Authors present a protocol for a scoping review aimed at assessing the role of different airway clearance techniques on ECMO.

The manuscript is well written, and deals with an important topic

I only have one minor comments for the Authors:

1. The Authors frequently discuss the issue of secretions clearance in patients with very low tidal volumes and airway pressures supported by ECMO. However, "awake" ECMO with or without invasive ventilation asl for respiratory failure is becoming increasingly popular (see https://pubmed.ncbi.nlm.nih.gov/37971332/; https://pubmed.ncbi.nlm.nih.gov/40500654/; https://pubmed.ncbi.nlm.nih.gov/39843275/; https://pubmed.ncbi.nlm.nih.gov/37723384/; https://pubmed.ncbi.nlm.nih.gov/39656144/). Please discuss the issue of airway clearance considering the possible application of awake ECMO

VERSION 1 - AUTHOR RESPONSE

Reviewer comments	Author response	Line number
Please clarify whether the person intervening is a doctor or another medical professional.	I have amended the concept section to clarify that we are interested in airway clearance delivered by any profession.<br><br>This is also referenced…..table 2	145-146, 196
Please consider interventions that can be performed by doctors (For example, bronchoscopy-assisted suction) and those that can	The scoping review aims to explore all airway clearance modalities. Bronchoscopy is	150-152

be performed by non-doctors (For example, squeezing by a physical therapist).	included as one of the listed interventions.	
Please distinguish whether the procedure was performed at an ECMO center that is thought to have an abundance of man-power or not (There is data that shows that ECMO treatment at ECMO centers improves patient outcomes, but this is said to be due to sufficient manpower allocation and the accumulation of sufficient case experience.)	I agree – staffing provision can impact on the intervention provided. To support this concept I have added an additional comment in the data extraction table (under clinician profession): "Staffing requirement for the delivered intervention (if provided)."	196
The Authors frequently discuss the issue of secretions clearance in patients with very low tidal volumes and airway pressures supported by ECMO. However, "awake" ECMO with or without invasive ventilation asl for respiratory failure is becoming increasingly popular.	Thank you for clarifying this key element. For the purpose of this scoping review I will be focussing solely on patients receiving invasive mechanical ventilation. These patients can be awake or sedated but must have ongoing invasive ventilation. I have made amendments to the introduction, PICO, Inclusion criteria and data extraction table.	13, 84, 89, 126, 175, 196
Tables should be in Word format and placed in the main text where the table is first cited. Please remove your table files, embedding them into the main text where the table is first cited. Tables should be created within the word document, not inserted as an image.	The separate table files have been removed, and the embedded versions retained within the main text. All tables are presented in Word format.	NA
Please provide a contact email address for your Ethics Committee or Institutional Board. If your work was not reviewed by an ethics committee, please provide a contact email address for the relevant Research Integrity department. Please provide details for the most suitable individual	The ethics statement has been revised to provide clarification regarding the absence of ethical approval requirements and to include information on	222-225

such as a head of department in the event of no integrity department.	local research governance oversight.		
